# Association between Initial Opioid Prescription and Patient Pain with Continued Opioid Use among Opioid-Naïve Patients Undergoing Elective Surgery in a Large American Health System

**DOI:** 10.3390/ijerph20105766

**Published:** 2023-05-09

**Authors:** Abass Babatunde, Lior Rennert, Kevin B. Walker, Douglas L. Furmanek, Dawn W. Blackhurst, Vito A. Cancellaro, Alain H. Litwin, Kerry A. Howard

**Affiliations:** 1 Department of Public Health Sciences, Clemson University, Clemson, SC 29634, USA; 2 Center for Public Health Modeling and Response, Clemson University, Clemson, SC 29634, USA; 3 Prisma Health, Greenville, SC 29605, USA; 4 School of Health Research, Clemson University, Clemson, SC 29634, USA; 5 School of Medicine Greenville, University of South Carolina, Greenville, SC 29605, USA

**Keywords:** pre-operative pain, post-operative pain, discharge pain, opioid doses, opioid-naïve, opioid prescription refills, patient-level factors

## Abstract

There is growing concern about the over-prescription of opioids and the risks of long-term use. This study examined the relationship between initial need (pre-operative, post-operative, and discharge pain) and dosage of opioids in the first prescription after surgery with continued opioid use through opioid refills over 12 months, while considering patient-level characteristics. A total of 9262 opioid-naïve patients underwent elective surgery, 7219 of whom were prescribed opioids following surgery. The results showed that 17% of patients received at least one opioid refill within one year post-surgery. Higher initial opioid doses, measured in morphine milligram equivalent (MME), were associated with a greater likelihood of continued use. Patients receiving a dose greater than 90 MME were 1.57 times more likely to receive a refill compared to those receiving less than 90 MME (95% confidence interval: 1.30–1.90, *p* < 0.001). Additionally, patients who experienced pain before or after surgery were more likely to receive opioid refills. Those experiencing moderate or severe pain were 1.66 times more likely to receive a refill (95% confidence interval: 1.45–1.91, *p* < 0.001). The findings highlight the need to consider surgery-related factors when prescribing opioids and the importance of developing strategies to balance the optimization of pain management with the risk of opioid-related harms.

## 1. Introduction

Opioids are frequently prescribed for pain management in the United States, where they are more commonly prescribed than in any other country [1]. However, the opioid epidemic has become a major public health concern in recent years, due to the alarming increase in opioid-related deaths [2]. Given the tendency for high doses of opioids to be used to control pain related to surgery, attention has turned to surgeons as a key population for opioid prescribing guidelines to curb the epidemic [3,4,5]. While it is well-established that prescription opioids for chronic pain put patients at risk of continued use, with the dosage of the initial prescription being significant, less is known about patients who are opioid-naïve, without chronic pain, who are first exposed to opioids as a product of elective surgery. Identifying characteristics related to opioid prescribing for elective surgeries that predict continued use may help to ascertain important factors for providers to consider when initially prescribing opioids to this patient population.

Prescribing opioids to opioid-naïve patients has been identified as a risk factor for continued use and opioid-related adverse events [6]. Even a short course of opioids can lead to prolonged use which can, in turn, lead to addiction and overdose [3,6,7,8,9,10,11]. Therefore, evaluating the practices of prescribing opioids, and their impacts on patients, particularly those who are opioid-naïve, is important. Factors that have been shown to contribute to opioid misuse and addiction include patient-related factors such as younger age, previous other substance use, and psychological disorders [6,12,13,14,15]. That being said, opioids are commonly prescribed for the management of post-operative pain, and failing to provide them when needed would cause unnecessary pain and suffering for patients [16]. Pain ratings are indicators of the need for pain management and are, therefore, additional characteristics related to the surgery and prescription of opioids. Further investigation into the balance between pain management and opioid dosage is necessary. A collective look at patient-level factors and factors related to surgery and pain management may help providers make informed decisions when initially prescribing opioids to this patient population.

The objective of this study was to examine the relationship between patient factors and factors related to the first opioid prescription post-surgery, including patient-reported pain, and continued opioid refills for opioid-naïve patients. The study aimed to investigate the impact of opioid prescription dosage, pre- and post-operative pain, and discharge pain on opioid refills in the 12 months following surgery in order to examine continued use. By understanding these factors, the findings of this study can inform clinical practice and guide the development of interventions to reduce the risk of opioid dependence and addiction in opioid-naïve patients who receive opioids for a surgical procedure.

## 2. Materials and Methods

### 2.1. Data Source and Patient Cohort

Prescription and surgery information for patients was extracted from Prisma Health’s Electronic Health Record (EHR) for the study period of April 2016 through September 2019. Prisma Health is the largest healthcare system in South Carolina, including 18 hospitals and 289 practice sites in Greenville and surrounding counties in the Upstate region.

**Figure 1:**
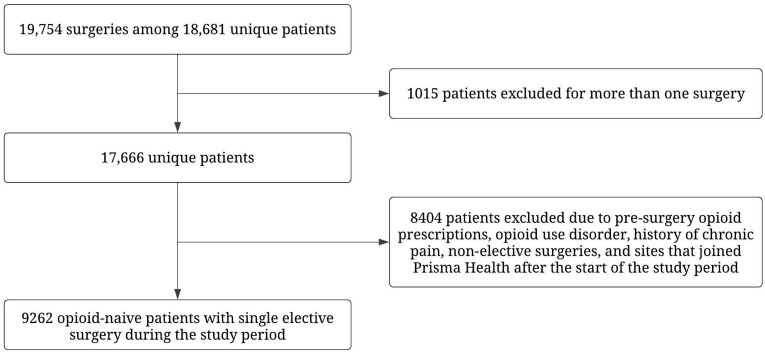
Diagram of surgeries and patients in the study period and those included in the analyses.

During the study period, 19,754 surgeries were performed in 16 Prisma Health facilities among 18,681 unique patients. A total of 1015 patients were removed from the analysis because they had more than one surgery during the study period. An additional 8404 patients were excluded due to pre-surgery opioid prescriptions, opioid use disorders, histories of chronic pain, non-elective surgeries, and surgeries performed at facilities that joined Prisma Health after the beginning of the study. Thus, the final sample consisted of 9262 opioid-naïve patients undergoing a single elective surgery during the study time (Figure 1). Surgeries were done under general anesthesia without added opioids.

### 2.2. Characteristics of the First Opioid Prescription and Need for Pain Management

The total daily opioid dosage in the first prescription post-surgery to patients in the cohort was calculated and recorded in morphine milligram equivalent (MME), taking into account all opioids. Standard calculation of MME is the product of the dose (strength multiplied by supply per day) and a conversion factor based on the opioid [17,18]. The prescription of interest was identified as the first prescription dated after the date of surgery and, therefore, would be filled by patients post-discharge. Participants were grouped into three categories: (1) 0 MME (no opioids prescribed), (2) 1–90 MME, and (3) greater than 90 MME prescribed during the first prescription post-surgery. A threshold of >90 MME/day was chosen because it has been used as an indicator of high-dose prescribing and generally recommended against [4,7,19,20,21]. Additionally, we examined patient-reported pain ratings. Patient-reported pain was recorded by nursing staff using categories of none, mild, moderate, or severe. Pain was reported prior to surgery, immediately following surgery, and at the time of discharge for pre-operative, post-operative, and discharge pain variables, respectively. Patients with pre-operative pain that required opioid-level pain management were given hydromorphone, but such patients were removed from the analysis for a pre-surgery opioid prescription. 

### 2.3. Data Analysis

We conducted logistic regression analyses to evaluate the relationship between predictors and the outcome of opioid refills within the 12-month period after surgery. The outcome variable was binary, indicating whether patients did or did not receive any opioid refills during the post-surgery period. The main predictors were MME/day prescribed in the first prescription post-surgery, pre- and post-operative pain, and discharge pain. MME dosage was categorized as greater than and less than or equal to 90, with subjects who did not receive any MME dosage removed from the analysis. The pain variables were binary and based on patient ratings, categorized as none/mild or moderate/severe pain for pre- and post-operative pain and discharge pain. Any pain was calculated based on if pre-operative, post-operative, or discharge pain was moderate/severe.

The effect of the main predictors (MME daily dosage and pain variables) was individually examined in the unadjusted model. In the adjusted model, we included age, sex, race/ethnicity, elective surgery category, mental health status, tobacco smoking, and tobacco vaping. These variables were included as covariates in the analysis. Statistical analyses were performed using R version 3.6, and we considered *p* < 0.05 as statistically significant. Ethical review and approval were waived for this study under Exemption Category 4.

## 3. Results 

The characteristics of the cohort are presented in Table 1. We identified 9262 opioid-naïve patients who underwent elective surgery at Prisma Health between April 2016 and September 2019. The mean age of the study cohort was 51.6 with a standard deviation of 16.3. The majority of patients identified as Non-Hispanic White (78.8%) and the majority identified as female (65.5%). The majority of the participants (62.7%) received MME > 90 in their first opioid prescription post-surgery. Another 15.2% received a dosage of MME ≤ 90, and 22.1% did not receive any MME in their first prescription post-surgery and were removed from further analyses. On average, 17% of patients who were prescribed any MME received any opioid prescription refill during the 12-month follow-up. There were more refills within the MME > 90 group (18.2%), while 10% of the MME ≤ 90 group got a refill. Pairwise comparisons of patient characteristics in the categories of 0 MME, MME between 0 and 90, and MME > 90 groups can be found in the Supplementary Materials. 

The results of the logistic regression analysis that examined the relationship between prescribed MME/day, discharge pain, pre- and post-operative pain, and opioid refills during the follow-up period are provided in Table 2. The unadjusted odds ratio (OR) for the MME > 90 group compared to the MME ≤ 90 group was 1.79 (95% confidence interval (CI) = 1.52–2.14; *p* < 0.001), indicating that those with MME > 90 were 1.79 times more likely to receive a refill than those in the MME ≤ 90 group. After adjusting for all other covariates and pain, the OR was still significant at 1.57 (95% CI = 1.30–1.90; *p* < 0.001).

Patients who reported severe or moderate pre-operative pain were 1.88 times more likely to get a refill in the follow-up period compared to those with none or mild pre-operative pain (95% CI = 1.64–2.16; *p* < 0.001), which was significant after adjusting for covariates (OR = 1.34; 95% CI = 1.10–1.62; *p* = *0*.003). Similarly, patients who had moderate or severe post-operative pain were almost twice as likely to get a refill compared to patients with none or mild pain after surgery (OR = 1.90; 95% CI = 1.66–2.16; *p* < 0.001); however, this did not show evidence of significance when adjusted for covariates (OR = 1.18; 95% CI = 0.97–1.43; *p* = 0.101). Those reporting discharge pain of moderate or severe levels were 1.76 times more likely to get a refill compared to those with none or mild discharge pain (95% CI: 1.55–2.00; *p* < 0.001), and this association remained significant after adjusting for other covariates (OR = 1.35; 95% CI: 1.16–1.57; *p* < 0.001). 

Patient-level characteristics were also predictive of likelihood of a refill. Non-Hispanic Black patients showed higher odds of receiving a refill compared to Non-Hispanic White patients (OR = 1.30; 95% CI = 1.07–1.54; *p* = 0.006); patients with psychological disorders showed higher odds of receiving a refill compared to those without (OR = 1.40; 95% CI = 1.22–1.61; *p* < 0.001); and current and former smokers showed higher odds of a refill compared to the non-smoker group, with odds ratios of 1.80 (95% CI = 1.51–2.15; *p* < 0.001) and 1.19 (95% CI = 1.01–1.40; *p* < 0.034), respectively. Full adjusted model results for patient-level characteristics can be found in Table 3.

## 4. Discussion

This study examined patient-level factors; initial need, based on pre-operative, post-operative, and discharge pain; and dosage of opioids in the first prescription after surgery and how these factors could affect continued opioid use in the form of opioid refills for opioid-naïve patients. In this cohort of 9262 opioid-naïve patients, 7219 (77.9%) were prescribed any MME in the first prescription following any elective surgery, with 1208 (17%) getting at least one refill within one year after the surgery. Furthermore, those with greater pain and a higher dosage of opioids showed greater likelihood of obtaining a refill. These overall findings suggest that opioid prescribing practices may contribute to the continued use of opioids, especially in patients receiving them for the first time, and tailoring prescriptions to individual patient needs should be considered. 

The results from our study demonstrated that the quantity of opioid dosage in the first prescription post-surgery is crucial and could impact the continued use of opioids. In particular, patients with an opioid dosage greater than 90 MME/day were 1.57 times more likely to have a refill compared to those who received a dosage of less than 90 MME. Therefore, the present study adds to a growing body of literature showing a link between opioid prescription and continued use [2,6,7,10,18,19,22,23,24,25]. That said, there is generally a lack of consistent guidelines for opioid prescribing post-surgery, particularly for opioid-naïve patients, with studies utilizing a variety of dosages (e.g., 50–2000 MME) or any dosage to examine the association between dose and later use [18,19,22,25,26]. Whereas 90 MME is considered high-dose prescribing, it is still lower than that examined in some studies, and our study detected a significant difference in the likelihood of a refill between ≤ 90 MME and >90 MME, demonstrating the need for guidelines for opioid-naïve patients [4,7,18,19,20,21,22]. Additionally, there has been a focus in the literature on opioid prescriptions for chronic or pre-operative pain and the risk of continued use, while surgical patients receive the greatest amount of potent opioids [6,7,10,19,23]. By contrast, our patients were opioid-naïve with no history of chronic pain. Therefore, these results offer novel insights into the association between initial opioid prescription dosage and the potential for continued use. 

Pain management is an essential factor that leads physicians to prescribe opioids. Long-term opioid use often begins with treating acute pain [27], with those who are prescribed higher quantities of opioids initially or refill their prescriptions at a greater risk of developing opioid addiction and overdose [28]. The results of our study show that patients who experienced pain before and after surgery were more likely to receive opioid refills. As a result, physicians need to consider the potential risks and benefits of opioid use when using it as part of comprehensive pain management, especially for opioid-naïve individuals. 

Alternative non-opioid medications and opioid prescribing protocols may be of use to minimize risks without jeopardizing pain management. To give some examples, a shift to use of ketamine instead of opioids during surgery, a decrease in the availability of opioid dosage vials, EHR notifications to alert prescribers to high doses, and limitations to the duration of post-surgery prescriptions have been shown to be effective at reducing the MME prescribed post-surgery without compromising patient pain [4]. However, in an evaluation of opioid-prescribing practices, such policies did not show an association with fewer refills of opioid prescriptions post-surgery [4]. Notably, refills themselves have been identified as a risk factor for further use over time for the patient or family members [26,29]. In that our study examined predictive factors for refills, rather than the association between refills and later use, the results inform prescribing behaviors that could proactively reduce the potential for this risk factor. Guidelines tend to focus on limiting the amount or duration of the initial prescription, but novel methods for limiting refill access may need to be assessed. 

Providers must also ensure that they do not produce unnecessary pain for patients by not prescribing opioids [16]. Given that the quantity of opioids initially prescribed and their continued use afterwards has been a topic of concern in recent years due to the ongoing opioid epidemic in the United States, many healthcare organizations have issued guidelines for the appropriate prescribing of opioids. For example, the Centers for Disease Control and Prevention recommends that healthcare providers prescribe the lowest effective dose of opioids for the shortest duration possible and avoid prescribing high doses [21]. However, because of the push for reducing opioid exposure through prescriptions, patients may continue to experience pain and look for alternative ways to get opioids besides the safety of a prescription [16,30]. This adds to the value and need for studies such as ours that examine surgery-related predictive factors in terms of need for opioids (pain) and exposure (opioid dosage in the first prescription) in order to balance need and the risk of harmful consequences. In addition to these factors, individualized tailoring may also need to be considered. Prior research has shown that risk for misuse and abuse can be higher based on patient-level factors. Our study was consistent with prior literature in demonstrating heightened likelihood of a refill for Non-Hispanic Black patients and patients with other substance use (smoking) and psychological disorders [6,12,13,14,15]. Furthermore, the odds of a refill based on higher dosage or pain was lower after adjusting for such patient-level factors. For example, there was not evidence that post-operative pain was predictive of odds of a refill in the adjusted model. Therefore, the likelihood of refills of opioid prescriptions after elective surgery is multifaceted, demonstrating the need for a comprehensive approach that is geared to the patient when considering the prescription of opioids post-surgery [22]. 

There are a few limitations to the present study that should be noted. First, the study was carried out in a single health system located in the Upstate region of South Carolina where the population is predominantly white. Thus, it may not be appropriate to apply our findings to other contexts. Second, we were unable to consider other variables that could have an impact on opioid prescription practices, including provider attributes and patient preferences. Furthermore, although we collected data on refills, we were not able to confirm whether patients actually used the prescribed opioids [31]. Similarly, we do not know the motivation behind obtainment of a refill. That is, it may be related to the initial pain, a new or alternative condition, or misuse. Finally, we are unable to conclude that the predictors explored here were a cause of refills. The predictive factors were associated with refills, but we do not have data suggesting that dosage, pain, or patient factors cause immediate or long-term misuse. Future studies should examine additional factors and timing of refills, including the duration of continued use that may stem from initial exposure to opioids through elective surgery (i.e., are patients getting refills a year after surgery), as well as which opioids are prescribed as a potential additional predictive factor for prescribers to consider. 

## 5. Conclusions

Given the risk of opioid misuse following surgery among patients previously opioid-naïve, characteristics of the surgery, including the patient’s need for pain management and MME prescribed, are necessary factors to consider in examining and preventing the threat of continued use. The present study provides evidence that high opioid prescription doses, pre-and post-operative pain, and discharge pain are associated with a higher likelihood of opioid refills in the 12 months following elective surgery in opioid-naïve patients. The results also showed that patient-level factors, including race, other substance use, and psychological disorders predicted likelihood of obtaining a refill. Strategies and guidance related to refill access are underexplored, particularly among opioid-naïve patients. The findings highlight the need for careful consideration of patient and surgery-related characteristics when prescribing opioids and the importance of developing strategies to balance optimization of pain management with risk of opioid-related harms. 

## Figures and Tables

**Table 1:** Characteristics of cohort.

Characteristics		Overall 9262	0 MME 2043 (22.1%)	0 < MME ≤ 90 1409 (15.2%)	MME >90 5810 (62.7%)	*p*-Value
Age, Mean (SD)		51.62 (16.34)	54.41 (16.48)	53.05 (16.84)	50.30 (16.02)	<0.001
Sex, *n* (%)	Male	3194 (34.5)	694 (34.0)	544 (38.6)	1956 (33.7)	0.002
	Female	6068 (65.5)	1349 (66.0)	865 (61.4)	3854 (66.3)	
Race/Ethnicity, *n* (%)	Hispanic	470 (5.1)	96 (4.7)	62 (4.5)	312 (5.4)	0.041
	Non-Hispanic White	7239 (78.8)	1641 (81.1)	1105 (79.7)	4493 (77.8)	
	Non-Hispanic Black	1315 (14.3)	257 (12.7)	190 (13.7)	868 (15.0)	
	Other	159 (1.7)	30 (1.5)	30 (2.2)	99 (1.7)	
Elective Surgeries (CPT Type), *n* (%)	Bariatric Surgery	31 (0.3)	22 (1.1)	0 (0.0)	9 (0.2)	<0.001
	Carpal Tunnel Release	797 (8.6)	383 (18.7)	185 (13.1)	229 (3.9)	
	Colectomy	568 (6.1)	39 (1.9)	54 (3.8)	475 (8.2)	
	Hemorrhoidectomy	380 (4.1)	51 (2.5)	13 (0.9)	316 (5.4)	
	Hysterectomy	1416 (15.3)	363 (17.8)	108 (7.7)	945 (16.3)	
	Laparoscopic Appendectomy	561 (6.1)	79 (3.9)	73 (5.2)	409 (7.0)	
	Laparoscopic Cholecystectomy	2359 (25.5)	452 (22.1)	348 (24.7)	1559 (26.8)	
	Parathyroidectomy	330 (3.6)	36 (1.8)	39 (2.8)	255 (4.4)	
	Reflux Surgery	51 (0.6)	7 (0.3)	14 (1.0)	30 (0.5)	
	Thyroidectomy	795 (8.6)	203 (9.9)	138 (9.8)	454 (7.8)	
	Transurethral Prostate Surgery	319 (3.4)	143 (7.0)	53 (3.8)	123 (2.1)	
	Ventral Hernia Repair	1655 (17.9)	265 (13.0)	384 (27.3)	1006 (17.3)	
Tobacco Smoking, *n* (%)	Current Smoker	1295 (14.0)	240 (11.7)	184 (13.1)	871 (15.0)	<0.001
	Former Smoker	2417 (26.1)	562 (27.5)	390 (27.7)	1465 (25.2)	
	Never Smoked	5526 (59.7)	1237 (60.5)	833 (59.1)	3456 (59.5)	
	Unknown	24 (0.3)	4 (0.2)	2 (0.1)	18 (0.3)	
Tobacco Vaping, *n* (%)	Current User	212 (2.3)	32 (1.6)	40 (2.8)	140 (2.4)	<0.001
	Former User	218 (2.4)	46 (2.3)	41 (2.9)	131 (2.3)	
	Never Used	7661 (82.8)	1661 (81.3)	1279 (90.8)	4721 (81.3)	
	Unknown	1167 (12.6)	304 (14.9)	49 (3.5)	814 (14.0)	
Mental Disorder, *n* (%)	Yes	3260 (35.2)	655 (32.1)	513 (36.4)	2092 (36.0)	0.003
	No	6002 (64.8)	1388 (67.9)	896 (63.6)	3718 (64.0)	
Pre-operative Pain, *n* (%)	None/Mild	6547 (70.7)	1411 (69.1)	1188 (84.3)	3948 (68.0)	<0.001
	Moderate/Severe	2044 (23.8)	400 (22.1)	187 (13.6)	1457 (27.0)	
Post-operative Pain, *n* (%)	None/Mild	6231 (67.3)	1353 (66.2)	1164 (82.6)	3714 (63.9)	<0.001
	Moderate/Severe	2333(27.2)	457 (25.2)	211 (15.3)	1665 (31.0)	
Discharge Pain, *n* (%)	None/Mild	6346 (68.5)	1501 (73.5)	1060 (75.2)	3785 (65.1)	<0.001
	Moderate/Severe	2916 (31.5)	542 (26.5)	349 (24.8)	2025 (34.9)	
MME in First Prescription, Mean (SD)		149.86 (155.60)	0.00 (0.00)	61.67 (14.84)	223.95 (152.56)	<0.001
Any Refill, *n* (%)	Yes	1209 (13.1)	--	149 (10.6)	1059 (18.2)	<0.001
	No	8053 (86.9)	--	1260 (89.4)	4751 (81.8)	

MME: Morphine milligram equivalents; SD: standard deviation; CPT: Current Procedural Terminology.

**Table 2:** Results from logistic regression analysis of relationship of MME prescribed and pain with refills.

	Unadjusted		Adjusted	
Predictors	Odds Ratio (95% CI)	*p*-Value	Odds Ratio (95% CI)	*p*-Value
MME > 90 (reference: MME ≤ 90)	1.79 (1.52–2.14)	<0.001	1.57 (1.30–1.90)	<0.001
Moderate/Severe Pre-operative Pain (reference: None/Mild)	1.88 (1.64–2.16)	<0.001	1.34 (1.10–1.62)	0.003
Moderate/Severe Post-operative Pain (reference: None/Mild)	1.90 (1.66–2.16)	<0.001	1.18 (0.97–1.43)	0.101
Moderate/Severe Discharge Pain (reference: None/Mild)	1.76 (1.55–2.00)	<0.001	1.35 (1.16–1.57)	<0.001
Any Pain (reference: No)	1.90 (1.67–2.96)	<0.001	1.66 (1.45–191)	<0.001

Any pain was calculated as moderate or severe pain among pre-operative, post-operative, or discharge pain. The adjusted analysis does not include other pain variables. CI: confidence interval.

**Table 3:** Full results of adjusted logistic regression for patient-level characteristics.

Predictors		Odds Ratio (95% CI)	*p*-Value
Age		1.00 (1.00–1.01)	0.951
Race/Ethnicity (reference: Non-Hispanic White)	Hispanic	1.00 (0.43–2.37)	0.916
	Non-Hispanic Black	1.30 (1.07–1.54)	0.006
	Non-Hispanic Other	1.43 (0.86–2.28)	0.151
Sex (reference: Male)	Female	1.15 (0.97–1.35)	0.102
Tobacco Smoking (reference: Never Smoked)	Current Smoker	1.80 (1.51–2.15)	<0.001
	Former Smoker	1.19 (1.01–1.40)	0.034
	Unknown	1.53 (0.34–5.03)	0.521
Tobacco Vaping (reference: Never Used)	Current User	1.00 (0.67–1.54)	0.988
	Former User	1.32 (0.72–2.35)	0.342
	Unknown	1.05 (0.67–1.69)	0.828
Psychological Disorder (reference: No)	Yes	1.40 (1.22–1.61)	<0.001

## Data Availability

Data can be made available upon reasonable request to the corresponding author.

## Supplementary Materials

The following supporting information can be downloaded at: https://www.mdpi.com/article/10.3390/ijerph20105766/s1, Table S1: Characteristics of the cohorts that received 0 MME and MME ≤ 90; Table S2: Characteristics of the cohorts that received 0 MME and MME > 90; Table S3: Characteristics of the cohorts that received 0–90 MME and MME > 90.
