# Supplementary material for: Association between Initial Opioid Prescription and Patient Pain with Continued Opioid Use among Opioid-Naïve Patients Undergoing Elective Surgery in a Large American Health System
Source: Int J Environ Res Public Health. 2023 May 9;20(10):5766. doi: 10.3390/ijerph20105766 (PMC10218665; doi:10.3390/ijerph20105766)
Supplement: Supplementary file 1 [file ijerph-20-05766-s001.zip › ijerph-2347006-supplementary.pdf]

Supplementary Materials

Table S1. Characteristics of the cohorts that received 0 MME and MME ≤ 90.

**Characteristics**	**0 MME**	**0 < MME ≤ 90**	***p*-value**
Age, Mean (SD)	54.41 (16.48)	53.05 (16.84)	**.002**
Sex, N (%)			**.006**
Male	694 (34.0)	544 (38.6)	
Female	1349 (66.0)	865 (61.4)	
Race/Ethnicity, N (%)			.202
Hispanic	96 (4.7)	62 (4.5)	
Non-Hispanic White	1641 (81.1)	1105 (79.7)	
Non-Hispanic Black	257 (12.7)	190 (13.7)	
Other	30 (1.5)	30 (2.2)	
Elective Surgeries (CPT type), N (%)			**<.001**
Bariatric Surgery	22 (1.1)	0 (0.0)	
Carpal Tunnel Release	383 (18.7)	185 (13.1)	
Colectomy	39 (1.9)	54 (3.8)	
Hemorrhoidectomy	51 (2.5)	13 (0.9)	
Hysterectomy	363 (17.8)	108 (7.7)	
Laparoscopic Appendectomy	79 (3.9)	73 (5.2)	
Laparoscopic Cholecystectomy	452 (22.1)	348 (24.7)	
Parathyroidectomy	36 (1.8)	39 (2.8)	
Reflux Surgery	7 (0.3)	14 (1.0)	
Thyroidectomy	203 (9.9)	138 (9.8)	
Transurethral Prostate Surgery	143 (7.0)	53 (3.8)	

Ventral Hernia Repair	265 (13.0)	384 (27.3)	
Tobacco Smoking, N (%)			.660
Current Smoker	240 (11.7)	184 (13.1)	
Former Smoker	562 (27.5)	390 (27.7)	
Never Smoked	1237 (60.5)	833 (59.1)	
Unknown	4 (0.2)	2 (0.1)	
Tobacco Vaping, N (%)			**<.001**
Current User	32 (1.6)	40 (2.8)	
Former User	46 (2.3)	41 (2.9)	
Never Used	1661 (81.3)	1279 (90.8)	
Unknown	304 (14.9)	49 (3.5)	
Mental Disorder, N (%)			**.009**
Yes	655 (32.1)	513 (36.4)	
No	1388 (67.9)	896 (63.6)	
Pre-operative Pain, N (%)			**<.001**
None/Mild	1411 (69.1)	1188 (84.3)	
Moderate/Severe	400 (22.1)	187 (13.6)	
Post-operative Pain, N (%)			**<.001**
None/Mild	1353 (66.2)	1164 (82.6)	
Moderate/Severe	457 (25.2)	211 (15.3)	
Discharge Pain, N (%)			.262
None/Mild	1501 (73.5)	1060 (75.2)	
Moderate/Severe	542 (26.5)	349 (24.8)	

Table S2. Characteristics of the cohorts that received 0 MME and MME > 90.

**Characteristics**	**0 MME**	**MME >90**	***p*-value**
Age, Mean (SD)	54.41 (16.48)	50.30 (16.02)	**<.001**
Sex, N (%)			.824
Male	694 (34.0)	1956 (33.7)	
Female	1349 (66.0)	3854 (66.3)	
Race/Ethnicity, N (%)			**.026**
Hispanic	96 (4.7)	312 (5.4)	
Non-Hispanic White	1641 (81.1)	4493 (77.8)	
Non-Hispanic Black	257 (12.7)	868 (15.0)	
Other	30 (1.5)	99 (1.7)	
Elective Surgeries (CPT type), N (%)			**<.001**
Bariatric Surgery	22 (1.1)	9 (0.2)	
Carpal Tunnel Release	383 (18.7)	229 (3.9)	
Colectomy	39 (1.9)	475 (8.2)	
Hemorrhoidectomy	51 (2.5)	316 (5.4)	
Hysterectomy	363 (17.8)	945 (16.3)	
Laparoscopic Appendectomy	79 (3.9)	409 (7.0)	
Laparoscopic Cholecystectomy	452 (22.1)	1559 (26.8)	
Parathyroidectomy	36 (1.8)	255 (4.4)	
Reflux Surgery	7 (0.3)	30 (0.5)	
Thyroidectomy	203 (9.9)	454 (7.8)	
Transurethral Prostate Surgery	143 (7.0)	123 (2.1)	
Ventral Hernia Repair	265 (13.0)	1006 (17.3)	

Tobacco Smoking, N (%)			**.002**
Current Smoker	240 (11.7)	871 (15.0)	
Former Smoker	562 (27.5)	1465 (25.2)	
Never Smoked	1237 (60.5)	3456 (59.5)	
Unknown	4 (0.2)	18 (0.3)	
Tobacco Vaping, N (%)			.132
Current User	32 (1.6)	140 (2.4)	
Former User	46 (2.3)	131 (2.3)	
Never Used	1661 (81.3)	4721 (81.3)	
Unknown	304 (14.9)	814 (14.0)	
Mental Disorder, N (%)			**.001**
Yes	655 (32.1)	2092 (36.0)	
No	1388 (67.9)	3718 (64.0)	
Pre-operative Pain, N (%)			**<.001**
None/Mild	1411 (69.1)	3948 (68.0)	
Moderate/Severe	400 (22.1)	1457 (27.0)	
Post-operative Pain, N (%)			**<.001**
None/Mild	1353 (66.2)	3714 (63.9)	
Moderate/Severe	457 (25.2)	1665 (31.0)	
Discharge Pain, N (%)			**<.001**
None/Mild	1501 (73.5)	3785 (65.1)	
Moderate/Severe	542 (26.5)	2025 (34.9)	

Table S3. Characteristics of the cohorts that received 0-90 MME and MME > 90.

**Characteristics**	**0 < MME ≤ 90**	**MME >90**	***p*-value**
Age, Mean (SD)	53.05 (16.84)	50.30 (16.02)	**<.001**
Sex, N (%)			**.001**
Male	544 (38.6)	1956 (33.7)	
Female	865 (61.4)	3854 (66.3)	
Race/Ethnicity, N (%)			**.003**
Hispanic	62 (4.5)	312 (5.4)	
Non-Hispanic White	1105 (79.7)	4493 (77.8)	
Non-Hispanic Black	190 (13.7)	868 (15.0)	
Other	30 (2.2)	99 (1.7)	
Elective Surgeries (CPT type), N (%)			**<.001**
Bariatric Surgery	0 (0.0)	9 (0.2)	
Carpal Tunnel Release	185 (13.1)	229 (3.9)	
Colectomy	54 (3.8)	475 (8.2)	
Hemorrhoidectomy	13 (0.9)	316 (5.4)	
Hysterectomy	108 (7.7)	945 (16.3)	
Laparoscopic Appendectomy	73 (5.2)	409 (7.0)	
Laparoscopic Cholecystectomy	348 (24.7)	1559 (26.8)	
Parathyroidectomy	39 (2.8)	255 (4.4)	
Reflux Surgery	14 (1.0)	30 (0.5)	
Thyroidectomy	138 (9.8)	454 (7.8)	
Transurethral Prostate Surgery	53 (3.8)	123 (2.1)	
Ventral Hernia Repair	384 (27.3)	1006 (17.3)	

Tobacco Smoking, N (%)			.078
Current Smoker	184 (13.1)	871 (15.0)	
Former Smoker	390 (27.7)	1465 (25.2)	
Never Smoked	833 (59.1)	3456 (59.5)	
Unknown	2 (0.1)	18 (0.3)	
Tobacco Vaping, N (%)			**<.001**
Current User	40 (2.8)	140 (2.4)	
Former User	41 (2.9)	131 (2.3)	
Never Used	1279 (90.8)	4721 (81.3)	
Unknown	49 (3.5)	814 (14.0)	
Mental Disorder, N (%)			.802
Yes	513 (36.4)	2092 (36.0)	
No	896 (63.6)	3718 (64.0)	
Pre-operative Pain, N (%)			**<.001**
None/Mild	1188 (84.3)	3948 (68.0)	
Moderate/Severe	187 (13.6)	1457 (27.0)	
Post-operative Pain, N (%)			**<.001**
None/Mild	1164 (82.6)	3714 (63.9)	
Moderate/Severe	211 (15.3)	1665 (31.0)	
Discharge Pain, N (%)			**<.001**
None/Mild	1060 (75.2)	3785 (65.1)	
Moderate/Severe	349 (24.8)	2025 (34.9)	
MME in first prescription, Mean (SD)	61.67 (14.84)	223.95 (152.56)	**<.001**
Any Refill, N (%)			**<.001**
Yes	149 (10.6)	1059 (18.2)	

No	1260 (89.4)	4751 (81.8)
